# Unmasking the Hidden Culprit: Diagnostic Challenges in Thoracic Spinal Osteomyelitis Following COVID-19 Infection

**DOI:** 10.7759/cureus.81816

**Published:** 2025-04-07

**Authors:** Kole P Joachim, Alina Katsman

**Affiliations:** 1 General Internal Medicine, University of California Los Angeles David Geffen School of Medicine, Westlake, USA

**Keywords:** ct-guided biopsy, infectious disease, spinal osteomyelitis, staphylococcus aureus, surgical debridement, thoracic vertebrae infection, vertebral osteomyelitis

## Abstract

Spinal osteomyelitis (SO) is a rare but serious infection of the vertebrae with occasional epidural involvement. Diagnosis is complicated by nonspecific findings that oftentimes mimic malignancy or tuberculosis, with typical methods of diagnosis such as blood culture and image-guided biopsy possessing varying levels of sensitivity. In this report, we present a rare case of SO that highlights these diagnostic difficulties. SO should be considered in a patient who presents with non-specific back pain. In such cases, non-diagnostic image-guided biopsy attempts do not rule out SO. Further investigations into additional diagnostic techniques to reduce false negatives are required to limit delays in diagnosis.

## Introduction

Spinal osteomyelitis (SO) is a serious bone infection, accounting for 3-5% of all osteomyelitis cases with an incidence of approximately 4.8 cases per 100,000 individuals in the United States [1]. The primary mechanism involves the hematogenous spread of bacteria commonly from urinary, respiratory, gastrointestinal, and soft tissue infections. Although less frequent, contiguous spread can occur from adjacent infections, such as decubitus ulcers, or through direct inoculation in spinal surgery [2]. Symptoms at presentation are frequently non-specific, with chronic back pain and fever being the most common findings. Neurological deficits such as paraplegia may also be seen in the setting of inappropriate or delayed treatment [2]. In addition, early diagnosis can be challenging, as constitutional symptoms oftentimes mimic malignancy or tuberculosis. Diagnosis relies on MRI, which typically demonstrates bone edema, abscesses, and epidural involvement, with a sensitivity of greater than 90% [3]. Given that blood cultures are only positive in 40-60% of all cases, pathogen identification often requires confirmation via CT-guided biopsy [4]. 

In this report, we describe a case of a patient who presented with a several-month history of thoracic back pain, night sweats, and weight loss following a COVID-19 infection. A subsequent CT chest angiogram revealed an infiltrative mass in the posterior mediastinum with a follow-up MRI demonstrating an infiltrating T4-T5 mass with epidural extension and severe canal stenosis. An attempt at CT-guided biopsy of the T4-T5 mass was equivocal, further complicating the diagnosis. Open biopsy and subsequent visualization of microbes on Grocott-Gomori methenamine silver and Giemsa stains confirmed a diagnosis of SO.

## Case presentation

**Figure 1:**
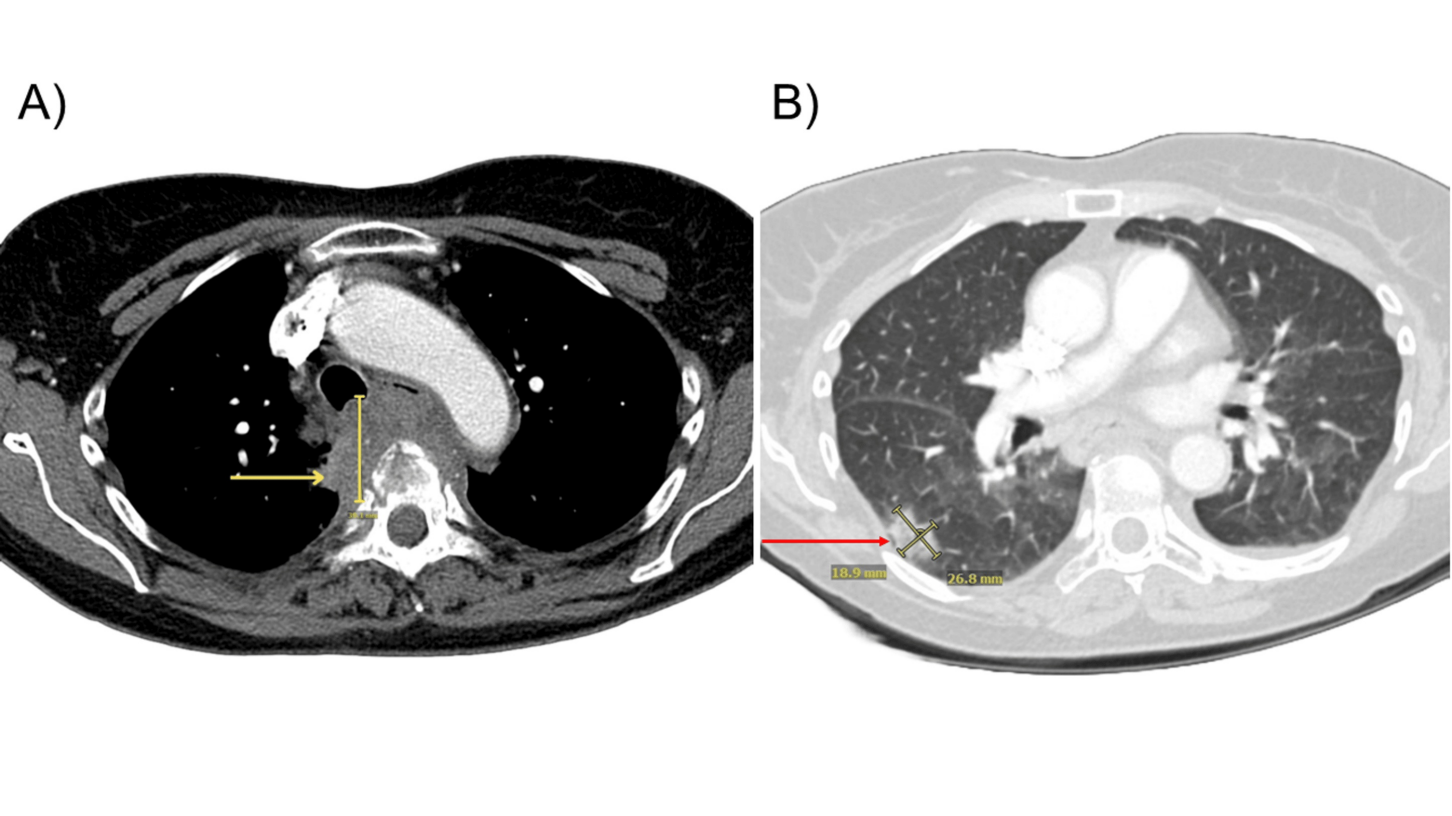
CT chest angiogram with contrast (A) Axial view from a contrast CT chest angiogram demonstrating an infiltrative soft tissue mass within the posterior mediastinum, with evidence of involvement of the adjacent vertebral bodies, suggestive of possible osseous invasion (noted by the yellow arrow). (B) Axial view of ground-glass nodular opacity noted in the right lower lobe, corresponding to an area of prior consolidation (noted by the red arrow). These findings raised concern for a possible underlying malignancy, particularly in the context of the patient's recent onset of thoracic back pain and persistent cough.

A 69-year-old female with no significant past medical history presented to her primary care physician 10 days after treatment for COVID-19 infection with Paxlovid. Two days prior to her presentation, the patient sought care at a local emergency department for acute onset shortness of breath and a two-day history of severe upper back pain, which she endorsed was worse at night and exacerbated by coughing. Laboratory studies from the emergency department were notable for an elevated D-dimer (0.74 mg/L FEU, age-adjusted D-dimer cutoff: 0.69 mg/L FEU) and B-natriuretic peptide (165.1 pg/mL, reference range: <100 pg/mL) (Table 1). The physical exam revealed absent peripheral edema, decreasing the likelihood of clinically overt heart failure at the time of presentation. A CT pulmonary angiogram (CTA) was subsequently performed, which was negative for pulmonary embolism but revealed a 2.3 mm consolidation in the right lower lobe and a 6 mm solid nodule in the right middle lobe with associated bronchiectasis. The finding of bronchiectasis prompted pulmonary function testing, which yielded normal spirometry and diffusion capacity results. Notably, she was a non-smoker with no previous pulmonary history or known environmental exposures at the time of imaging and spirometry testing. Two days later, she presented to her primary care physician with ongoing thoracic back pain and a persistent cough. She was subsequently prescribed budesonide/formoterol, referred to pulmonology, and instructed to repeat a CTA in six weeks for interval follow-up of the previously noted consolidation and to ensure resolution. The CTA was performed as planned and revealed a new infiltrative mass in the posterior mediastinum involving the adjacent vertebral bodies with ground glass nodular opacity in the right lower lobe not seen on prior imaging concerning for possible malignancy (Figure 1).

**Table 1:** Laboratory values obtained at the time of presentation to the emergency room following the development of acute-onset shortness of breath and upper back pain. FEU: fibrinogen equivalent units

Test	Baseline values (one month prior)	Results (emergency room)	Reference range
Hemoglobin	12.7	12.7	13.3-17.7 g/dL
White blood cell count	3.28	7.48	4.5-11.0 k/uL
Platelets	206	237	150-440 k/uL
Mean corpuscular volume	88.2	86.4	80-99 fL
Red cell distribution width	13.8	13.3	12-15%
Glucose	97	118 (H)	74-106 mg/dL
Calcium	9.7	9.2	8.5-10.01 mg/dL
Total Bilirubin	0.3	0.6	0.2-1.0 mg/dL
Aspartate transaminase (AST)	27	18	15-37 U/L
Alanine transaminase (ALT)	22	23	13-56 U/L
Albumin	4.6	3.9	3.4-5.0 g/dL
B-natriuretic peptide	N/A	165.1 (H)	<100 pg/mL
Troponin-I	N/A	6.0	3.0-53.9 pg/mL
D-dimer, quantitative	N/A	0.76 (H)	<0.50 mg/L FEU

**Figure 2:**
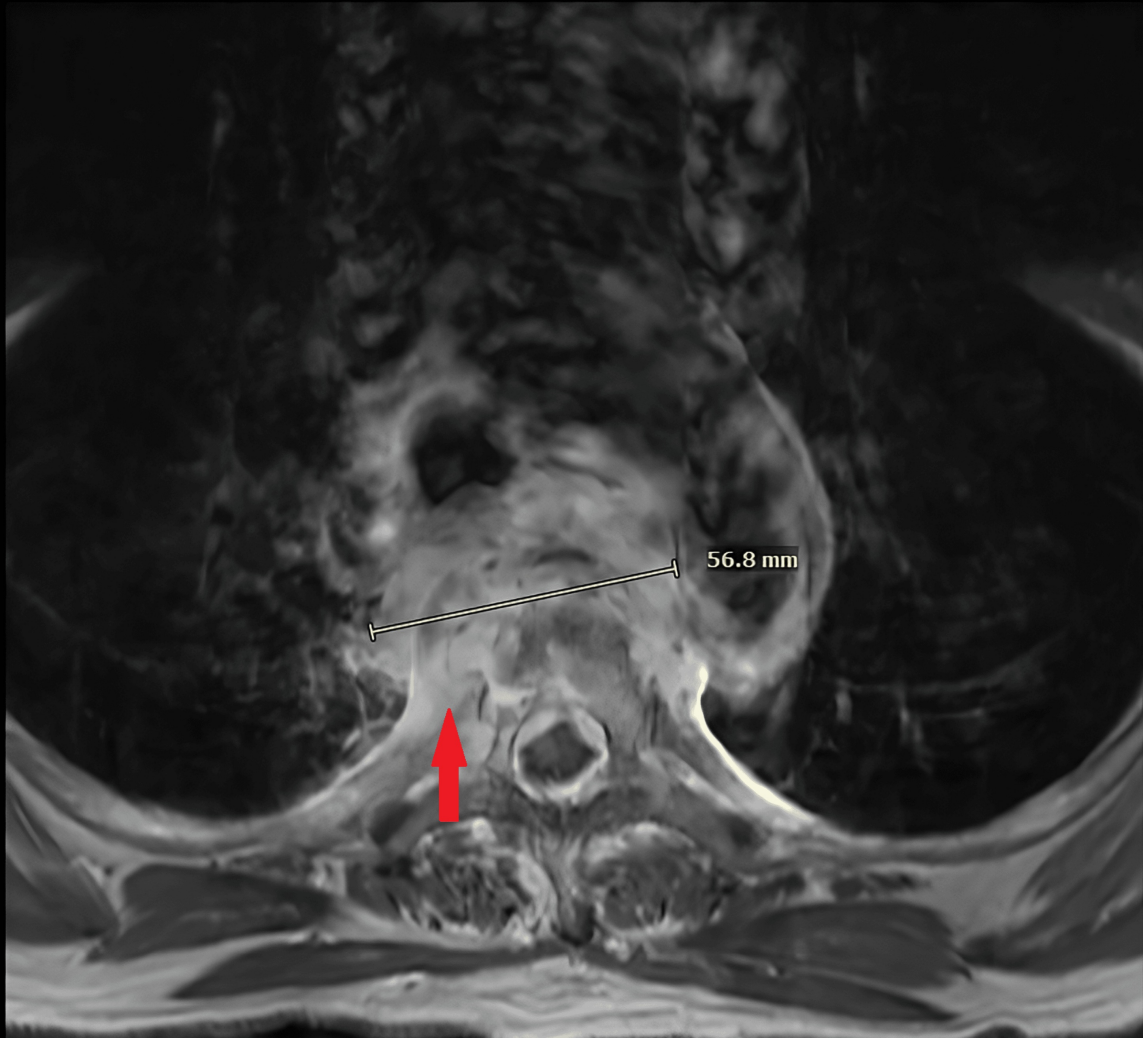
Multisequence MRI of the thoracic spine obtained with intravenous contrast Axial view obtained from the MRI of the thoracic spine revealing an enhancing infiltrative mass centered at the T4 and T5 vertebral bodies with associated endplate irregularity and extension into the adjacent epidural space (noted by red arrow). The imaging characteristics, including vertebral body involvement and epidural extension, raised concern for spinal osteomyelitis

Considering the concerning CTA findings, further evaluation was warranted to better characterize the extent and nature of the lesion. As a result, an MRI of the thoracic spine with and without contrast was obtained to assess for spinal cord involvement and epidural extension and to help differentiate between malignant, infectious, or inflammatory processes. The results of the MR spine illustrated an infiltrating and enhancing mass centered at the T4 and T5 vertebral bodies with epidural extension, suggesting possible spinal osteomyelitis or sclerosing mediastinitis (Figure 2). To identify possible infectious etiology, a percutaneous CT-guided coaxial core needle biopsy was performed, which yielded nondiagnostic results but demonstrated granulation and fibrous tissue with patchy mixed acute and chronic lymphoplasmacytic inflammation. A consult was placed to Infectious Disease, who recommended that the patient present for an expedited workup of the mediastinal mass with microbial analysis. Coccidioides IgG and IgM serologies, *Mycobacterium tuberculosis* QuantiFERON, cryptococcal antigen, rapid plasma reagin, HIV serology, and two sets of blood cultures were obtained, which all yielded negative results. Further workup with bronchoscopy and endobronchial ultrasound (EBUS) biopsy were negative for malignant cells but remained non-diagnostic with findings suspicious for sclerosing mediastinitis and possible infection.

**Figure 3:**
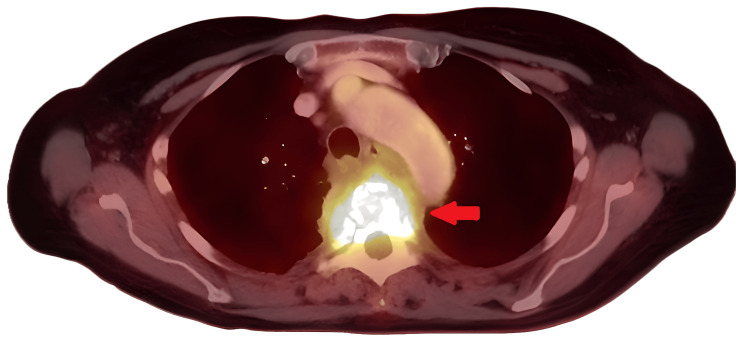
PET CT with a diagnostic CT of the neck, chest, abdomen, and pelvis with IV contrast; FDG Axial positron emission tomography/computed tomography (PET/CT) image demonstrating intense 18F-fluorodeoxyglucose (FDG) uptake (noted by the red arrow) centered at the T4–T5 disc space, with associated destruction of the adjacent vertebral bodies and extension into the posterior mediastinum and spinal canal. These findings correspond to a metabolically active, infiltrative lesion consistent with spinal osteomyelitis with imaging features suggestive of an infectious or granulomatous process.

**Figure 4:**
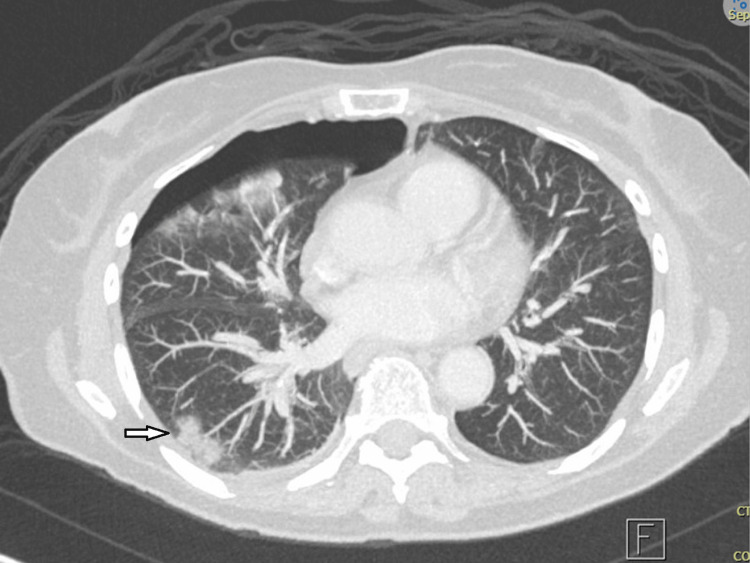
Ground-glass nodular opacity in the right lower lobe (noted by the outlined arrow)

Given the inconclusive results from both the CT-guided biopsy and bronchoscopy with EBUS-despite persistent suspicion for either an infectious or inflammatory etiology such as sclerosing mediastinitis or spinal osteomyelitis, further diagnostic clarity was needed. With a broad infectious workup, including serologies and blood cultures returning negative, and malignancy not definitively excluded, a PET/CT scan was subsequently performed to better characterize the metabolic activity of the thoracic spine mass, assess for additional sites of involvement, and help differentiate between infectious, inflammatory, and neoplastic processes. The PET/CT scan demonstrated an FDG-avid soft tissue mass centered at the T4-T5 disc space with extension into the posterior mediastinum and spinal canal, accompanied by progressive destruction of the T4 and T5 vertebral bodies-findings that strongly favored an infectious etiology such as granulomatous disease, though malignancy could not be entirely excluded (Figure 3). Additional notable findings included a small to moderate right pneumothorax, likely related to recent bronchoscopy; a persistent ground glass nodular opacity in the right lower lobe, stable from prior imaging and of uncertain significance; and intense FDG uptake with wall thickening in the nondistended cecum, raising the possibility of physiologic activity versus an underlying neoplastic process (Figure 4).

Given the unexpected finding of intense FDG uptake with wall thickening in the nondistended cecum on PET/CT-raising concern for a possible neoplastic process, a colonoscopy was performed to further evaluate for underlying colorectal pathology. This diagnostic step was taken to clarify the nature of the cecal activity and to rule out synchronous malignancy. The results of the colonoscopy revealed tubular adenomas and no high-grade dysplasia. In parallel, attention remained focused on the thoracic spine lesion, which continued to raise concern given its associated vertebral destruction, epidural extension, and suspected infectious etiology. Neurosurgery was consulted due to thoracic spinal cord compression with associated myelopathy. She subsequently underwent T3-7 posterior spinal fusion, T5 corpectomy, and L4/5 laminectomy. Intraoperative findings were notable for purulence at the T4-T5 level and a mediastinal abscess located ventral to the vertebral body, which was thoroughly debrided and irrigated. Bacterial cultures grew methicillin-sensitive Staphylococcus aureus (MSSA). Based on these findings, Infectious Disease recommended treatment with cefadroxil 1 g twice daily, along with a four-week course of linezolid and an eight-week course of rifampin. Given the confirmed MSSA mediastinal infection with epidural space involvement and vertebral osteomyelitis, cefadroxil was continued for long-term suppression.

Following recovery from neurosurgical intervention and initiation of targeted antibiotic therapy, attention returned to the persistent ground glass opacity in the right lower lobe, which had previously demonstrated stability but remained of uncertain etiology. Given the lesion’s unchanged appearance over multiple imaging studies and the prior differential that included indolent malignancy, a repeat CT chest was performed three months postoperatively to re-evaluate the nodule. The CT revealed a nodule that remained unchanged from what was previously demonstrated in Figure 4. A biopsy was performed and revealed adenocarcinoma (moderately to poorly differentiated, acinar pattern with complex glandular growth, EGFR negative, ALK negative, PD-L1 < 1%). The patient subsequently underwent a successful right lower lobectomy and lymph node dissection. Lymph nodes were negative for malignancy, and the patient was subsequently determined to have stage IA2 (pT1bN0) non-small cell lung cancer with no further indications for adjuvant chemotherapy or radiation.

## Discussion

This report highlights the diagnostic challenges associated with the presentation of SO. Blood cultures from the patient yielded no growth, and an attempt at image-guided biopsy of the infiltrating T4-T5 mass was equivocal. This aligns with recent studies reporting that the rate of positive blood cultures in SO varies from 30% to 89%, while the yield of CT-guided percutaneous sampling ranges from 31% to 91% [5]. This is in contrast to open biopsy that has a yield of approximately 76% to 91% [6]. While image-guided biopsy is oftentimes the initial method of choice given its lower complication rates and hospitalization time, its lack of sensitivity can lead to delayed diagnosis, as in this patient [6]. In addition, the mediastinal mass present in this patient warranted further evaluation by endobronchial ultrasound biopsy (EBUS) to exclude possible neoplasms, which also yielded indeterminate results. While open biopsy results ultimately identified visible microbes on Grocott-Gomori methenamine silver and Giemsa stains, the absence of microbial growth from culture and repeated non-diagnostic image-guided biopsies highlights the limitations of relying on traditional microbiological methods alone. Consequently, there has been emerging evidence that new technologies such as shotgun metagenomic sequencing (sMGS) for detecting microbial cell-free DNA (mcfDNA) might be able to assist in diagnosing vertebral osteomyelitis, with preliminary studies showing an improvement in diagnostic accuracy by 11.6% over traditional invasive diagnostic approaches [7]. Continued advances in diagnostic technologies such as mcfDNA have led to a push for a multi-modal approach to reduce diagnostic delays.

There are certain comorbidities that are associated with an increased risk of developing SO. For example, diabetic patients possess a 4.2-fold higher risk of developing SO compared to those without diabetes. Immunosuppression in the setting of malignancy and chronic corticosteroid use also pose as risk factors, oftentimes presenting as atypical pathogens such as Mycobacterium tuberculosis or Brucella. In rare cases, fungal SO might occur dependent on clinical context with Aspergillus fumigatus common in immunocompromised patients and Candida often presenting in those with indwelling catheters. Staphylococcus aureus is the most commonly reported microorganism overall and comprises approximately 42-84% of isolated organisms, depending on the study population [8-12]. In addition, coagulase-negative staphylococci and Streptococcus species have an incidence of approximately 8.9% and 13.6%, respectively [2]. Our patient had no significant comorbidities at the time of presentation; however, she was later diagnosed with early-stage lung adenocarcinoma, which may have contributed to a degree of immune dysregulation. This aligns with the understanding that Staphylococcus aureus can affect individuals with subtle or evolving risk factors, making it a plausible pathogen even in patients without overt immunosuppression or chronic illness at the time of infection.

Treatment options for SO are dependent on microbial origin. Broad coverage with vancomycin 0.5-2.0 grams/day IV is typically initiated while awaiting blood culture and pathology results, followed by a tailored antibiotic course. In the setting of suspected hematogenous osteomyelitis without vertebral involvement, piperacillin-tazobactam 4.5 g IV q6h is added for empiric coverage against *Enterobacteriaceae* species and *Pseudomonas aeruginosa*. However, in vertebral osteomyelitis, ceftriaxone 2 g IV q12hr is oftentimes used in place of piperacillin-tazobactam. In the setting of vancomycin allergy or intolerance, patients may be switched to linezolid 600 mg IV q12hr [13]. As with our patient, rifampin therapy might be instituted for Staphylococcus aureus native SO as studies have demonstrated an approximate 14% decrease in the risk of clinical failure. In the setting of methicillin-susceptible *Staphylococcus aureus* (MSSA) infection with epidural space involvement, cefazolin might be administered in place of antistaphylococcal penicillins due to lower risk of mortality and a similar risk of recurrent infection [14]. As with our patient, completion of an adequate cefazolin regimen is often followed by cefadroxil as a long-term oral option [15]. 

Long-term patient follow-up is necessary in cases of vertebral osteomyelitis with recurrence rates estimated at around 14% [16]. Factors contributing to recurrence include inadequate antibiotic courses, incomplete surgical debridement, and lack of control of contributing comorbidities. In addition, methicillin-resistant *Staphylococcus aureus* infections have a higher incidence of recurrence than those with MSSA. After four weeks of an appropriate antibiotic regimen, the Infectious Disease Society of America recommends monitoring C-reactive protein and erythrocyte sedimentation rate to assess for clinical improvement. A follow-up MRI is suggested in the scenario of a lack of improvement in inflammatory markers, but not recommended in patients that demonstrate clinical and laboratory responses to therapy [17]. 

## Conclusions

This case highlights a diagnostically challenging presentation of SO with associated epidural abscess and mediastinal extension. Despite nondiagnostic results from both CT-guided biopsy and EBUS, persistent clinical concern prompted further workup with PET/CT, which revealed intense FDG uptake at T4-T5 and ultimately warranted neurosurgical intervention. Intraoperative findings confirmed purulence, and cultures grew MSSA, establishing a definitive diagnosis of bacterial SO and mediastinal abscess. Treatment required surgical debridement, spinal stabilization, and prolonged antimicrobial therapy. This case underscores the limitations of early biopsy in vertebral infections, the value of advanced imaging for guiding diagnosis, and the importance of multidisciplinary collaboration. It also demonstrates the potential for concurrent pathologies, as the patient was later diagnosed with early-stage lung adenocarcinoma, reinforcing the need for comprehensive evaluation when multiple lesions are present on imaging.
